# Identification of new loci for salt tolerance in soybean by high-resolution genome-wide association mapping

**DOI:** 10.1186/s12864-019-5662-9

**Published:** 2019-04-25

**Authors:** Tuyen D. Do, Tri D. Vuong, David Dunn, Michael Clubb, Babu Valliyodan, Gunvant Patil, Pengyin Chen, Dong Xu, Henry T. Nguyen, J. Grover Shannon

**Affiliations:** 1 Division of Plant Sciences, University of Missouri, Columbia, MO 65211 USA; 2 Division of Plant Sciences, University of Missouri, Portageville, MO 63873 USA; 3 Department of Electric Engineering and Computer Science, Christopher S. Bond Life Sciences Center, University of Missouri, Columbia, MO 65211 USA; 4 Present address: The Cuu Long Delta Rice Research Institute, Thoi Lai District, Can Tho City, Vietnam; 5 Present Address: Department Agronomy and Plant Genetics, University of Minnesota, St. Paul, MN 55108 USA

**Keywords:** Soybean [*Glycine max* (L.) Merr*.*)], Salt tolerance, Genome-wide association study (GWAS), Single nucleotide polymorphism (SNP), Gene-based marker, Candidate genes

## Abstract

**Background:**

Salinity is an abiotic stress that negatively affects soybean [*Glycine max* (L.) Merr.] seed yield. Although a major gene for salt tolerance was identified and consistently mapped to chromosome (Chr.) 3 by linkage mapping studies, it does not fully explain genetic variability for tolerance in soybean germplasm. In this study, a genome-wide association study (GWAS) was performed to map genomic regions for salt tolerance in a diverse panel of 305 soybean accessions using a single nucleotide polymorphism (SNP) dataset derived from the SoySNP50K iSelect BeadChip. A second GWAS was also conducted in a subset of 234 accessions using another 3.7 M SNP dataset derived from a whole-genome resequencing (WGRS) study. In addition, three gene-based markers (GBM) of the known gene, *Glyma03g32900*, on Chr. 3 were also integrated into the two datasets. Salt tolerance among soybean lines was evaluated by leaf scorch score (LSS), chlorophyll content ratio (CCR), leaf sodium content (LSC), and leaf chloride content (LCC).

**Results:**

For both association studies, a major locus for salt tolerance on Chr. 3 was confirmed by a number of significant SNPs, of which three gene-based SNP markers, Salt-20, Salt14056 and Salt11655, had the highest association with all four traits studied. Also, additional genomic regions on Chrs. 1, 8, and 18 were found to be associated with various traits measured in the second GWAS using the WGRS-derived SNP dataset.

**Conclusions:**

A region identified on Chr. 8 was identified to be associated with all four traits and predicted as a new minor locus for salt tolerance in soybean. The candidate genes harbored in this minor locus may help reveal the molecular mechanism involved in salt tolerance and to improve tolerance in soybean cultivars. The significant SNPs will be useful for marker-assisted selection for salt tolerance in soybean breeding programs.

**Electronic supplementary material:**

The online version of this article (10.1186/s12864-019-5662-9) contains supplementary material, which is available to authorized users.

## Background

In the worldwide crop agriculture, salinity is considered a major abiotic stress. The global extent of salt-affected soils amounts to 1128 million ha, of which 60% are saline soils, 26% are sodic soils, and 14% are saline-sodic soils [1]. Among irrigated agricultural land, an estimated 60 million ha is affected by salinity worldwide [2], and 0.25–0.5 million ha annual salt build-up causes significant loss of agricultural production [3]. The United States has large areas of soils with 5.2 million ha [2] or approximately 23% of the total irrigated land that is salt affected. Exploiting plant salinity tolerance has been shown to be among the effective strategies to limit losses from naturally occurring salinity and from the threat of human activities coupled with global climate changes [2, 4, 5].

Although soybean is classified as a moderately salt tolerant crop plant with a threshold of 5 dS/m, salt sensitive soybean cultivars were severely affected under salt stress and did not produce seeds at a soil salinity level of 8 dS/m [6, 7]. Soybean yield losses could result from reduced germination, low seedling emergence, and poor plant growth and development [8–11]. In addition, soybean seed protein, oil and carbohydrate content are negatively affected by salinity [12, 13].

Bi-parental quantitative trait locus (QTL) mapping was successfully implemented to identify and confirm a major locus on chromosome (Chr.) 03 for salt tolerance in soybean [14–20]. The candidate gene (*Glyma03g32900*) underlying salt tolerance was identified on Chr. 3 and related to a sodium transporter [17, 19, 21]. Near-isogenic lines carrying the salt tolerant gene were selected using molecular markers and showed high yield under saline field conditions [21, 22]. Gene-based makers (GBM) were developed for marker-assisted selection (MAS) and also for identifying new tolerance genes [20, 23]. Minor loci for salt tolerance were also identified on chromosomes (Chrs.) 2, 7, 9, 11, 13, 14, 15 and 18 [20, 24, 25]. In addition, other soybean sources may carry new gene(s) for salt tolerance [15, 19, 23]. Nevertheless, the major limitations of bi-parental linkage mapping can detect alleles from parents only and a few recombination events occur in mapping populations [26].

Genome-wide association studies (GWAS) present some advantages over linkage mapping among individuals in natural populations to exploit broader genetic diversity [27–29]. The concern for GWAS is spurious associations (false positive or Type I error) that can be caused by population stratification and cryptic relatedness [30–33]. However, various statistical procedures have been developed to reduce and control this issue. For instance, a Mixed Linear Model (MLM) with incorporation of population structure and a kinship matrix effectively eliminated false positives in GWAS [34, 35]. Multiple-Locus Linear Mixed Model (MLMM) was then developed based on MLM by adding significant markers as covariates in a stepwise MLM to remove the confounding effect between testing markers and relatedness [36, 37]. The MLMM, with the advantage in controlling false positives, has been successful for association mapping in *Arabidopsis*, common wheat, rice, pea, sorghum, and tomato [38–43].

Recently, many plant genomes have been re-sequenced using next-generation sequencing (NGS) technologies. The exploration of whole-genome re-sequencing (WGRS) data was considered as one of the requirements for GWAS [44]. Sequence-based GWAS was successfully applied for mapping agronomic traits and identifying the candidate genes inside of significant genomic regions in rice, peach, and foxtail millet [45–47]. In a GWAS study in rice, in addition to the confirmation of the reported genes, new genes were identified for agronomic traits using WGRS data. The results of confirming those new genes by overexpression were highly accurate [48]. Although SNP chip data was successfully used in GWAS to provide valuable genetic information, the higher density of SNP data generated from WGRS would be more precise in determining the candidate genes controlling traits of interest [45, 48].

An association mapping study for salt tolerance was first reported in soybean by analyzing seed germination rate under salt conditions [49]. The study identified three genomic regions significantly associated with the ratio of imbibition rate, the ratio of germination index, and the ratio of germination rate under salt conditions and mapped to Chrs. 8, 9, and 18 [49]. The major locus for salt tolerance on Chr. 3 mapped by bi-parental linkage mapping was confirmed using SoySNP50K iSelect BeadChip and WGRS data of 106 soybean lines [23]. In addition to the major locus on Chr. 3, eight additional genomic regions significantly associated with both leaf chloride concentrations and leaf chlorophyll concentrations were mapped on Chrs. 2, 7, 8, 10, 13, 14, 16, and 20 using SoySNP50K iSelect BeadChip dataset and were recommended for future studies [50].

In this study, two SNP marker datasets, the SoySNP50K iSelect BeadChip accessed from the Soybase (*http://www.soybase.org*) and the WGRS-derived 3.7 M SNPs, which were developed in the Soybean Genetics and Genomics Lab (Valliyodan and Nguyen, unpublished data), along with three salt tolerant GBMs [20, 23] were used for association mapping of salt tolerance among soybean plant introductions (PIs) from a core collection (Drs. Cregan and Song, pers. comm.). The objectives of this study were to map additional loci for salt tolerance other than the known locus on Chr. 3 and to identify new sources of salt tolerance for genetic analysis and breeding to improve salt tolerance in soybean.

## Results

### Phenotypic variation, heritability and correlation of salt tolerance traits

**Fig. 1:**
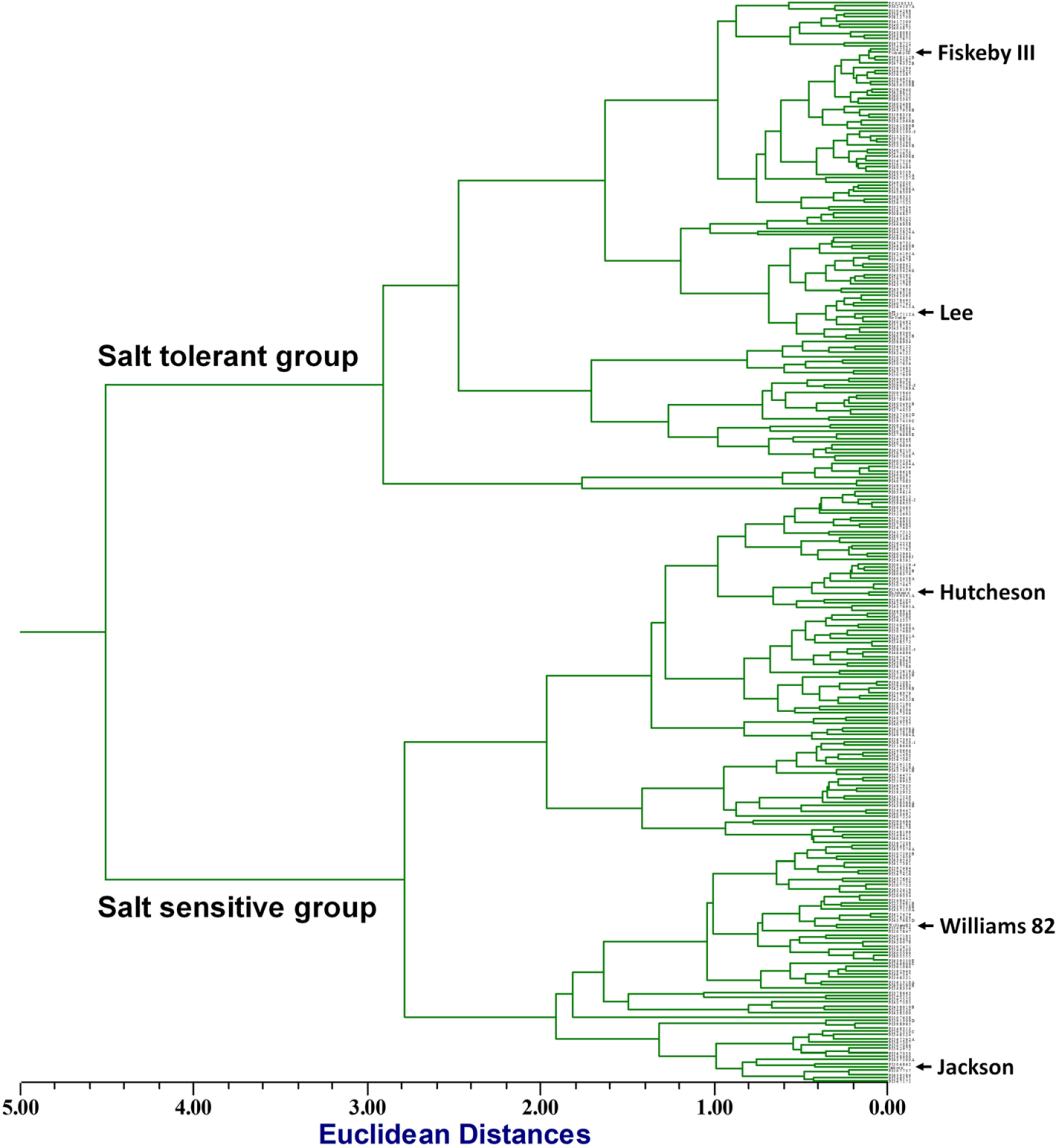
Phenotypic dendrogram for salt tolerance variation among 305 soybean accessions. This was constructed by combining leaf scorch score (LSS), chlorophyll content ratio (CCR), leaf sodium content (LSC) and leaf chloride content (LCC)

Two salt-sensitive, Hutcheson and Jackson, and two salt-tolerant, Lee and Fiskeby III, checks were clustered into corresponding groups as shown in phenotypic tree (Additional file 1: Table S1; Additional file 2: Figure S1; Fig. 1). The phenotypic variation among 305 soybean lines was statistically significant for all traits studied, including leaf scorch score (LSS), chlorophyll content ratio (CCR), leaf sodium content (LSC) and leaf chloride content (LCC), ranging from 1 to 5, 0.3–1.2, 0.03–1.7 (g kg^− 1^) and 2.7–18.2 (g kg^− 1^), respectively (Table 1). Some soybean lines evaluated showed higher salt tolerance than the salt-tolerant checks and some soybean lines were more sensitive to salt than the sensitive checks.

**Table 1:** Statistics for salt tolerance traits of a diverse panel of 305 soybean accessions

Trait	Min^a^	Max^b^	Mean	Root MSE^c^	CV^d^ (%)	H^2e^	Pearson correlation		
							CCR	LSC	LCC
LSS	1.0	5.0	2.7	0.52	18.9	0.8	−0.922**	0.638**	0.743**
CCR	0.3	1.2	0.8	0.04	5.9	0.9		−0.606**	− 0.700**
LSC (g kg^− 1^)	0.03	1.70	0.45	0.31	76.98	0.29			0.692**
LCC (g kg^− 1^)	2.70	18.20	7.85	1.74	22.21	0.63			

*LSS* leaf scorch score, *CCR* chlorophyll content ratio, *LSC* leaf sodium content, *LCC* leaf chloride content

**: Correlation coefficients are significant at the 0.01 level (2-tailed)

^a^: minimum; ^b^: maximum; ^c^: square root of mean square error; ^d^: coefficient of variation; ^e^: Broad-sense heritability

Correlation coefficients and heritability were estimated for the four traits in the diverse panel. The Pearson correlations indicated a significant linear relationship among the four traits at α = 0.01. Among these, correlation coefficients (*r*^*2*^) of CCR with the other traits were negative and ranged from −0.92 to −0.61. Conversely, the correlations between LSS, LSC and LCC were positive (Table 1). The broad-sense heritability was estimated based on analysis of variance for all traits (Table 1). The lowest value of heritability was 0.29 for LSC while higher heritability was observed in LSS, CCR and LCC as 0.82, 0.94 and 0.63, respectively. These results indicate that all four measurements are valid and representative of salt tolerance and can be used in screening and selection for salt tolerance. However, LSC and CCR appear to be more feasible and efficient in a practical breeding program.

In addition, the variation among 305 soybean accessions was shown by combining the four traits to construct a phenotypic dendrogram into salt-tolerant and salt-sensitive groups. A dissimilarity matrix that measures the relative distance of each pairs in salt tolerance of soybean lines showed a range of Euclidean distance from 0.06 to 10.81. The clustering analysis for the Euclidean distance matrix in the dendrogram revealed two main groups of soybean lines at the lowest Euclidean value and subgroups at higher values. Thereby, 137 soybean lines, including salt-tolerant checks belonged to salt-tolerant group and the remaining lines, including salt-sensitive checks were grouped into salt-sensitive group (Fig. 1).

### Linkage disequilibrium decay, population structure and relatedness among soybean accessions

Linkage disequilibrium (LD) decay, population structure and relatedness were analyzed for the panel of 305 soybean accessions using 37,573 SNPs of the SoySNP50K dataset and for the subset of 234 accessions using over 3.7 M SNPs of the WGRS-derived SNP dataset. An average distance between two markers was approximately 29.36 kb for the SoySNP50K dataset and 0.43 kb for the WGRS-derived SNP dataset. LD decay on all adjacent SNP pairs are in nonlinear curves (Additional file 3: Figure S2) with the LD blocks at an *r*^*2*^ of 0.2 with 293.64 and 371.42 kb for the SoySNP50K and the WGRS-derived SNP datasets, respectively. Thus, the number of SNPs was sufficient to cover the genome-wide haplotype blocks for both datasets.

**Fig. 2:**
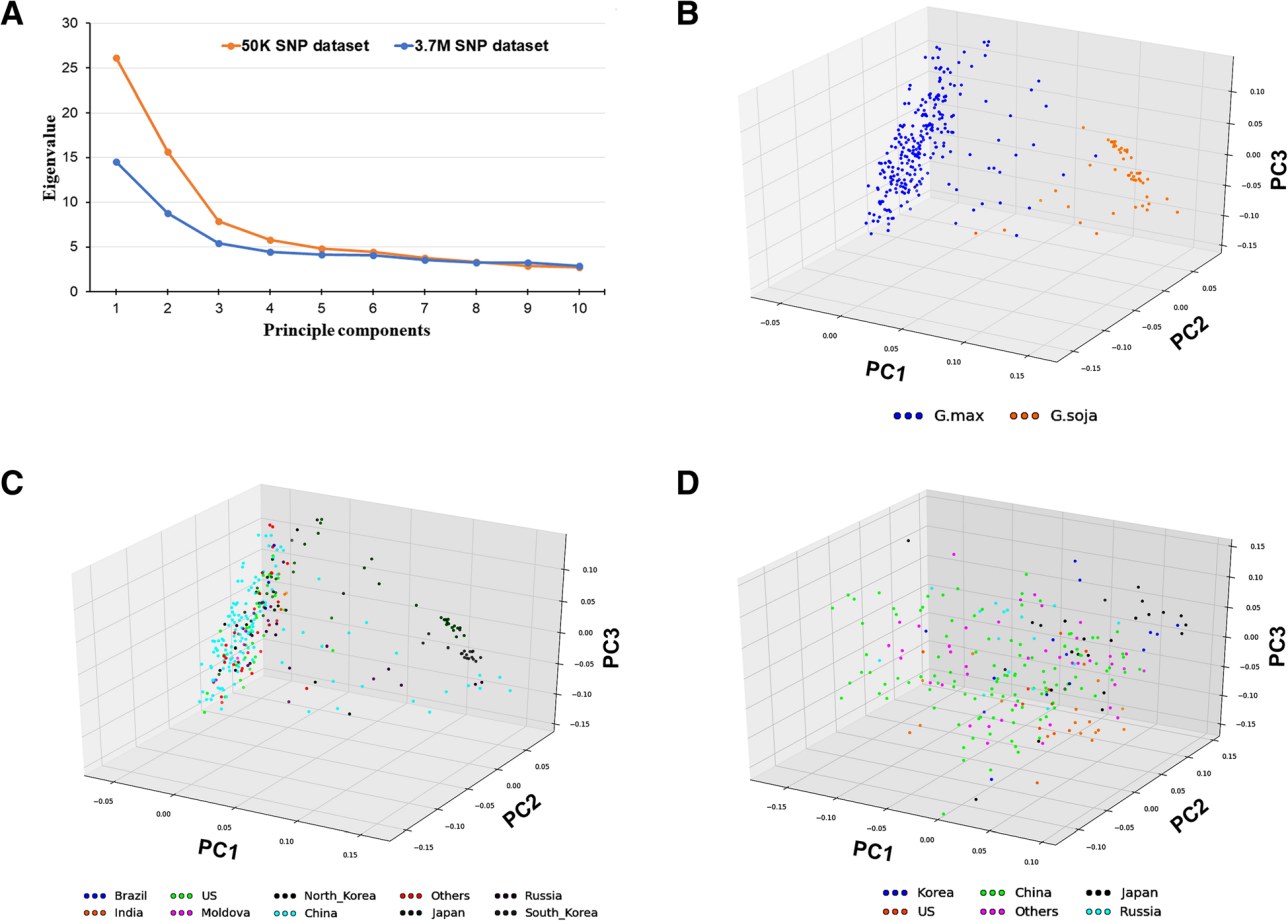
Population structure by principal component analysis (PCA) of two SNP datasets. **a** Screen plot of the first 10 principal components (PCs) and their contribution to Eigenvalue by analyzing SoySNP50K dataset (*yellow*) and 3.7 M SNP dataset (*blue*). **b** 3D scatterplot showed the first three PCs from SoySNP50K dataset corresponding to taxonomic groups for 305 soybean accessions. **c** 3D scatterplot showed the first three PCs from SoySNP50K dataset corresponding to original groups of 305 soybean accessions. **d** 3D scatterplot showed the first three PCs from 3.7 M SNP dataset corresponding to 234 soybean accessions selected from the original 305 accessions

The principal component analysis (PCA) showed that the variance explained, by eigenvalue of each PC, rapidly dropped after the first 4 PCs for both SNP datasets (Fig. 2a). The cumulative eigenvalues of the first three PCs were 49.7 and 28.7% of variances for the diverse panel and the subset using the SoySNP50K and the WGRS-derived SNP datasets, respectively. For the diverse panel using 37,573 SNPs, 305 soybean accessions were separated into groups roughly corresponding to taxonomy (*G. max* and *G. soja*) and country of origin (Figs. 2b and c) according to the first three PCs. However, the subset of 234 *G. max* soybean accessions was less defined into groups based on the first three PCs using over 2.2 M SNPs (Fig. 2d).

**Fig. 3:**
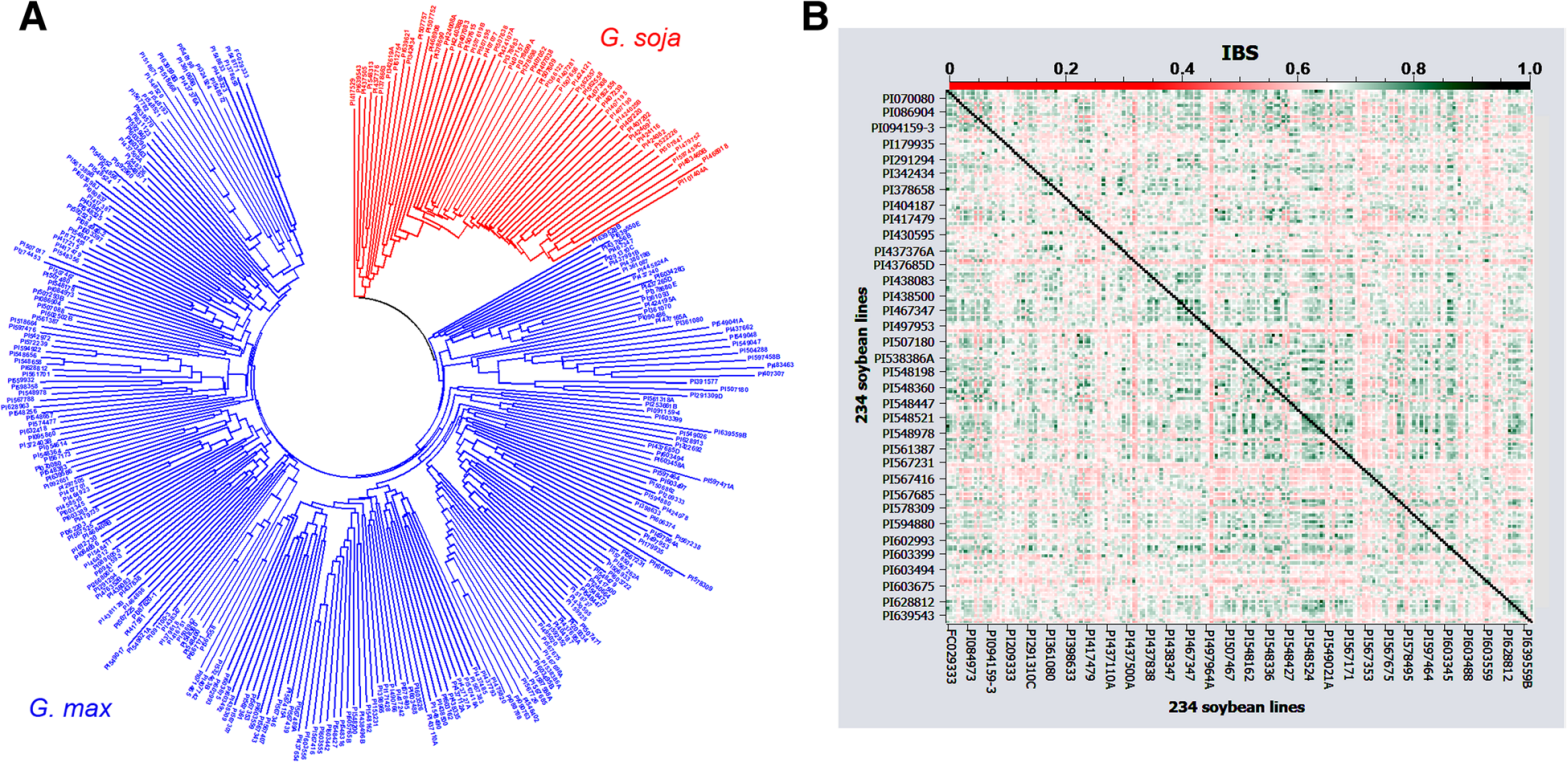
Relationships among soybean accessions using SoySNP50K and 3.7 M SNP datasets. **a** Phylogenetic tree of 305 soybean accessions using SoySNP50K dataset. **b** Heatmap plot showing the relationship among 234 soybean accessions using 3.7 M SNP dataset

The cryptic relatedness among soybean accessions was evaluated by kinship matrix from identity by state (IBS) each from paired soybean lines. The matrix with a range of IBS from 0.42 to 0.97 was calculated using the SoySNP50K dataset for the diverse panel to construct a phylogenetic tree showing the relationship among 305 soybean accessions (Fig. 3a). In this dendrogram, two main groups, *G. max* and *G. soja*, were clustered at the lowest IBS value and there were subgroups at higher IBS values. In a similar analysis, the kinship matrix with a narrower range of IBS from 0.52 to 0.89 was found by analyzing the WGRS-derived SNP dataset in the subset. The relatedness among 234 *G. max* soybean accessions was also shown in a heat map constructed from these IBS matrices (Fig. 3b).

The genomic inflation factors (lambda,λ) from association tests were applied to verify correction for population stratification by including PCs and kinship in the GWAS models (Additional file 4: Table S2). The lambda values from association tests for all traits were high using the general linear model (GLM) without correction for stratification with ranges of 2.21–3.95 and 1.44–2.08 by analyzing the SoySNP50K iSelect BeadChips and the WGRS-derived SNP datasets, respectively. The lower and closer to one values with a range of 1.05–1.20 were calculated with a population correction by PCs in P models. By adding more kinship matrices in EMMAX (Efficient mixed-model association expedited) and MLMM (Multi-locus mixed model) models, the pseudo-lambda values were from 0.93 to 1.03 for association of all traits from both genetic data sets indicating the GWAS results were not inflated by population structure or cryptic relatedness.

### GWAS for salt tolerance of the diverse panel using the SoySNP50K dataset

**Fig. 4:**
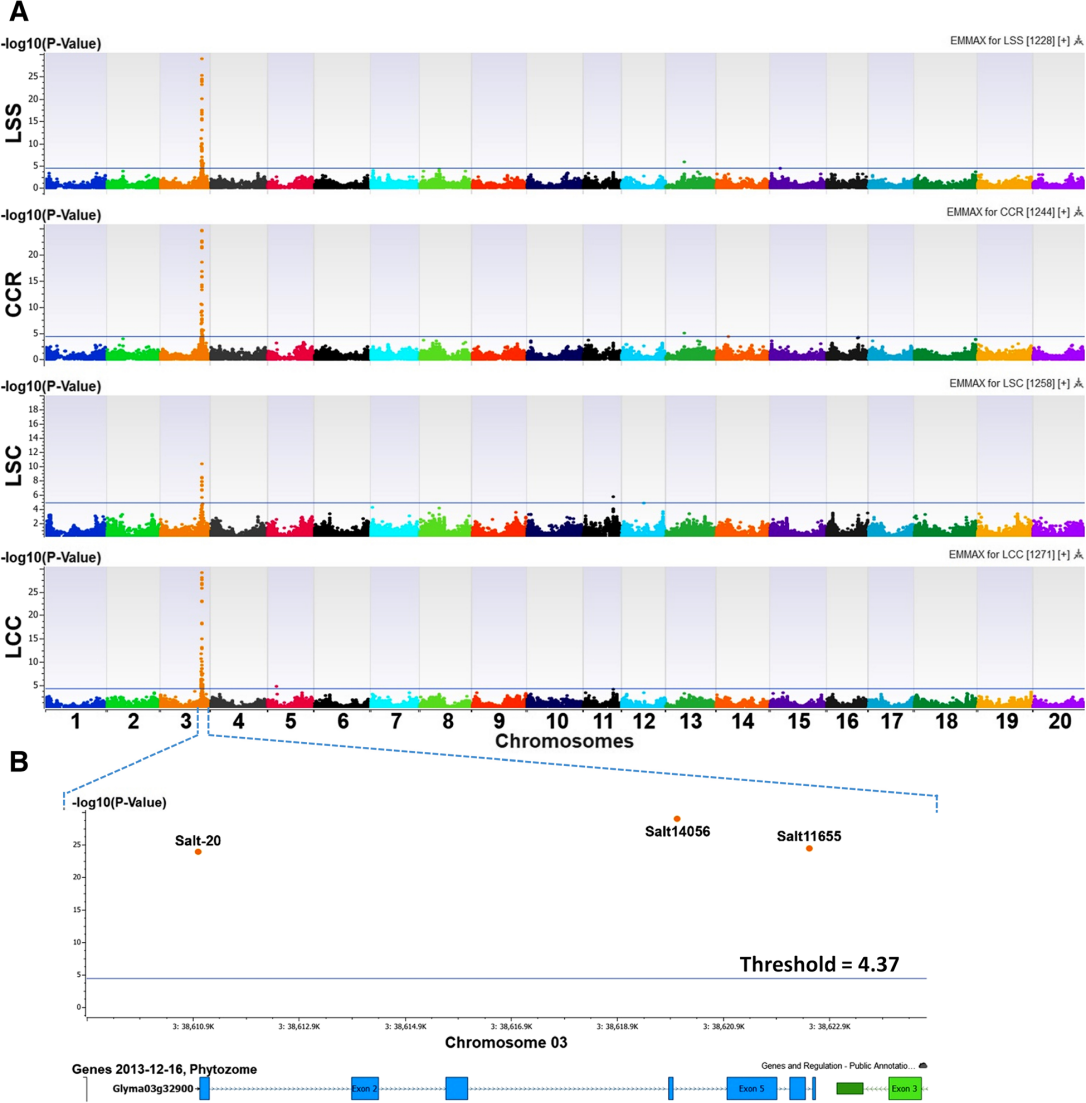
Association mapping of salt tolerance using EMMAX with a SoySNP50K dataset for 305 diverse soybean accessions. **a** Manhattan plots showed association of SNPs distributed throughout 20 chromosomes with leaf scorch score (LSS), chlorophyll content ratio (CCR), leaf sodium content (LSC) and leaf chloride content (LCC). **b** Three GBMs associated with salt tolerance and their position inside the known gene (*Glyma03g32900*), named in SoyBase Wm82 Genome Browser version 1

After marker quality control and assurance, a total of 37,573 polymorphic SNPs were selected from the SoySNP50K dataset and were utilized for further analysis of LSS, CCR, LSC, and LCC in the diverse panel. The quantile-quantile (Q-Q) distributions showed most SNPs matched what was expected (Additional file 5: Figure S3). The inflation values (Additional file 4: Table S2), the EMMAX and MLMM with corrections for the population stratification from the kinship matrix and the three first PCs were optimal for all four traits evaluated in this study. Numbers of SNPs significantly associated with LSS, CCR, LSC, and LCC were 44, 38, 13, and 54 based on corrections Benjamini-Hochberg of False Discovery Rate (FDR) ≤ 0.05. SNPs surrounding the known salt tolerance gene on Chr. 3 were the most significant in all four traits (Table 2; Fig. 4b). In addition, several SNPs significantly associated with one or two traits were identified on other chromosomes using the EMMAX analysis such as ss715616720 on Chr. 13 significantly associated with LSS and CCR, ss715609949 on Chr. 11 and ss715611871 on Chr. 12 with LSC, ss715592375 on Chr. 5 with LCC, and ss715592375 on Chr. 15 with LSS (Table 2; Fig. 4a). However, those significant SNPs, except the GBMs, were not detected after step 1 of MLMM.

**Table 2:** Several SNPs significantly associated with salt tolerance evaluated in GWAS using the SoySNP50K dataset for 305 soybean accessions

Marker	Chr.	Position	-log10(*P*)				Call Rate (%)	MA	MAF	Allele Refer	Cloned/Candidate genes ^a^
			LSS	CCR	LSC	LCC					
Salt-20	3	38,610,964	23.9*	22.3*	7.2*	25.7*	99.7	C	0.40	C/G	*Glyma03g32900* ^b^
Salt14056	3	38,619,995	28.9*	24.7*	10.3*	29.1*	100	G	0.44	C/G	*Glyma03g32900* ^b^
Salt11655	3	38,622,492	24.4*	22.5*	7.8*	27.9*	99.7	T	0.41	G/T	*Glyma03g32900* ^b^
ss715592375	5	7,534,622	2.5 ns	3.1 ns	2.4 ns	4.7*	98.0	G	0.22	A/G	No gene
ss715609949	11	27,743,052	3.8 ns	2.6 ns	5.6*	4.1 ns	99.0	A	0.06	G/A	No gene
ss715611871	12	20,568,054	2.6 ns	2.5 ns	4.8*	3.3 ns	99.3	T	0.10	G/T	No gene
ss715616720	13	16,871,244	5.8*	4.9*	1.8 ns	3.2 ns	99.7	T	0.31	C/T	No gene
ss715623199	15	9,138,970	4.4*	2.2 ns	1.0 ns	1.3 ns	100	T	0.24	C/T	*Glyma.15 g116200*
-log10(*P*) threshold ^c^			4.4	4.3	4.8	4.2					

*LSS* leaf scorch score, *CCR* chlorophyll content ratio, *LSC* leaf sodium content, *LCC* leaf chloride content (LCC)

*: Significant association with salt tolerant traits; ns: None significant association with trait

^a^: The candidate gene in Soybase Wm82 Genome Browser version 2 consist of significant SNP; ^b^: The cloned gene for salt tolerance is named *Glyma03g32900* in SoyBase Wm82 Genome Browser version 1; ^c^: Threshold was calculated based on *P*-value using False Discovery Rate correction (Benjamini-Hochberg); MA: Minor allele; MAF: Minor allele frequency

### GWAS for salt tolerance of the subset using the WGRS-derived SNP dataset

The subset of 234 *G. max* soybean accessions with 3.7 M SNPs derived from the WGRS project (*http://www.soybase.org*) was chosen to further perform GWAS for salt tolerance. To ensure that population size had no effect on GWAS results, this subset was analyzed separately by reusing SoySNP50K dataset. Similar results were found compared to GWAS of salt tolerance using SoySNP50K dataset for the diverse panel of 305 soybean lines. However, only the major locus on Chr. 3 was detected (Additional file 6: Figure S4).

**Fig. 5:**
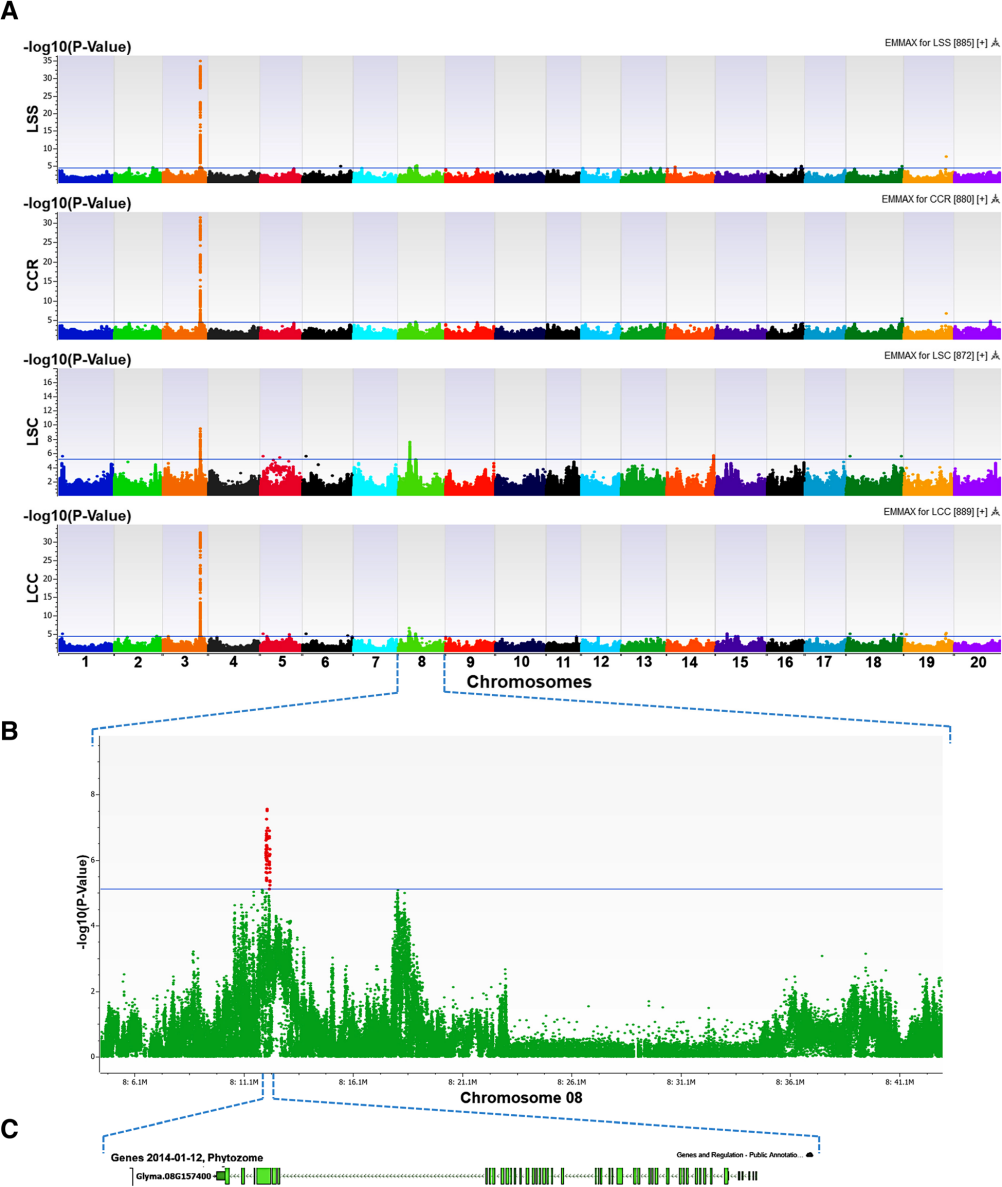
Association mapping of salt tolerance using EMMAX with 3.7 M SNP dataset for the subset of 234 soybean accessions. **a** Manhattan plots showed association of SNPs distributed throughout 20 chromosomes with leaf scorch score (LSS), chlorophyll content ratio (CCR), leaf sodium content (LSC), and leaf chloride content (LCC). **b** The significant genomic region associated with LSC on Chr. 8. **c** One of the pu tative candidate gene underlying a minor locus for salt tolerance

**Fig. 6:**
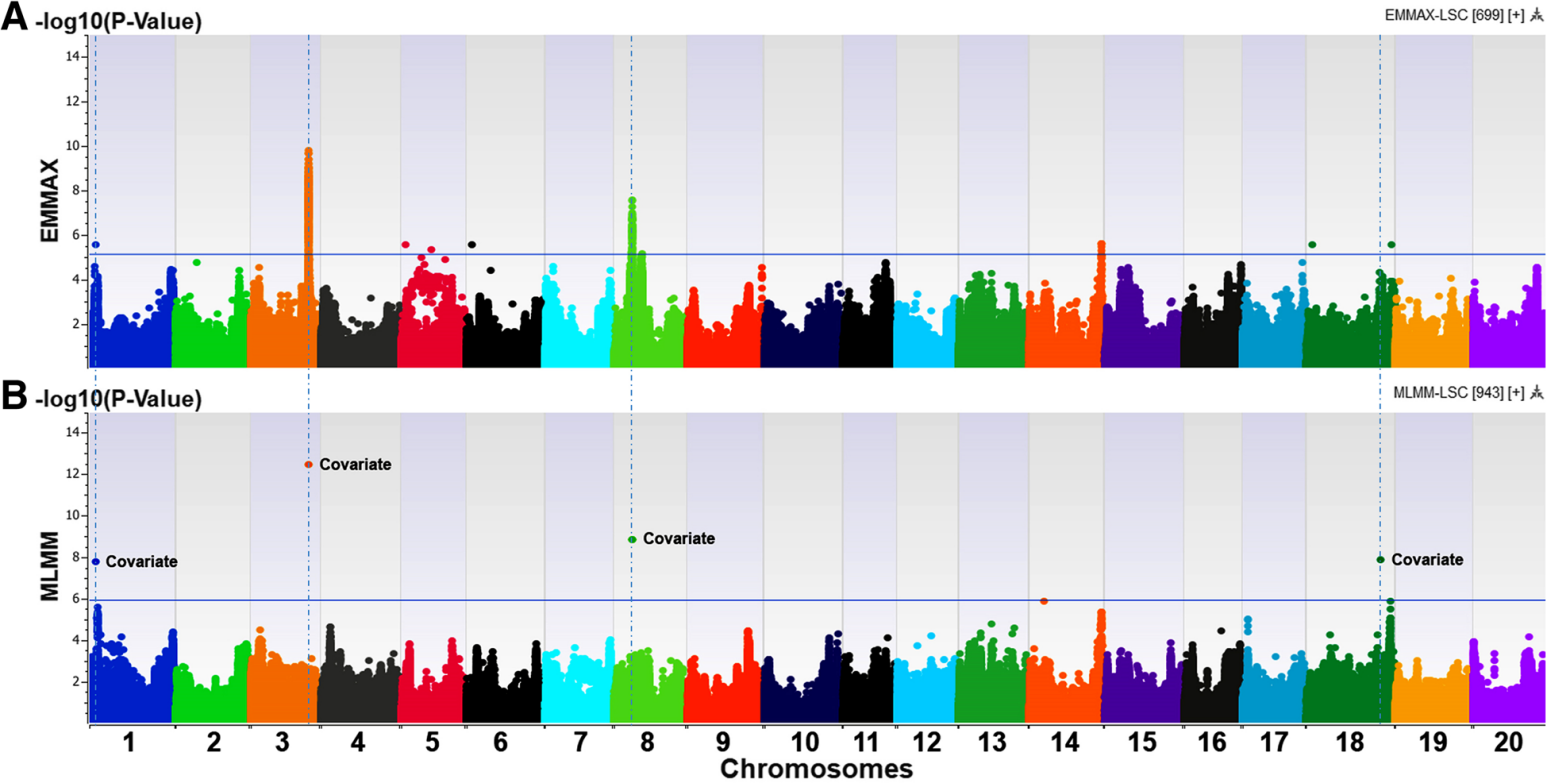
Comparison between EMMAX and MLMM in GWAS for salt tolerance. **a** Manhattan plot showed association of SNPs with leaf sodium content (LSC) by sequence-based GWAS using EMMAX. **b** Manhattan plot showed covariates (the most significant SNP after each step in stepwise analysis) associated with LSC by sequence-based GWAS using ML

The SNPs derived from the WGRS project were subjected to further quality control and assurance with MAF > 0.05 and the call rate > 0.95. Over 2.2 M polymorphic SNPs were obtained for further analysis. This dataset was employed for LD calculation, kinship construction, PCA, genotype association tests, and subsequently for GWAS of the four salt tolerant traits. Based on the Q-Q distributions most SNPs matched according to expectations (Additional file 7: Figure S5). In GWAS mixed models, the inflation values (Additional file 4: Table S2), with six PCs as a fixed effect and the kinship matrix as a random effect, were optimal for controlling confounding risk due to population stratification. The numbers of SNPs significantly associated with LSS, CCR, LSC and LCC from the EMMAX model analysis were 217, 190, 136 and 278, respectively, based on a FDR correction (Benjamini-Hochberg) with FDR ≤ 0.05 and selecting causal SNPs surrounding the know gene on Chr. 3. The significant SNPs associated with salt tolerant traits were located on Chrs. 1, 2, 3, 5, 6, 8, 14, 15, 16, 18, 19 and 20 (Fig. 5a) and the most significant SNPs on each chromosome are listed in Table 3. SNPs inside the known gene for salt tolerance with the highest -log10 (*P*) values, including three GBMs on Chr. 3, were significantly associated with all of four traits. Genomic regions on Chrs. 8 and 18 were also significantly associated with LSS, CCR, LSC and LCC, of which two adjacent peaks were in the significant region on Chr. 8. In addition, GWAS for salt tolerance using MLMM indicated that the significant positions in Chrs. 1, 3, 8 and 18 genomic regions were added as covariates (the most significant SNP after each step in the stepwise analysis) in the stepwise analysis (Fig. 6). Results show, the positions on Chr. 3 and Chr. 8 appeared to be associated with all of 4 salt tolerant traits while the positions on Chr. 1 and Chr. 18 were covariates in GWAS for LSC and LCC. Thus, the genomic region on Chr. 8 (Fig. 5B) is suggested as a minor locus for salt tolerance in addition to the major gene on Chr. 3 and the other significant genomic regions associated with individual traits measured to detect salt tolerance in this study.

**Table 3:** The most significant SNPs on each chromosome associated with salt tolerance traits detected in an association analysis using the WGRS-derived SNP dataset in the subset of 234 soybean accessions

Marker	Chr.	Position	-log10(*P*)				Call Rate (%)	MA	MAF	Allele Refer	Cloned/Candidate genes ^a^
			LSS	CCR	LSC	LCC					
1:4591993-SNV	1	4,591,993	1.1 ns	2.3 ns	5.5*	4.9*	100	T	0.50	T/C	No gene
2:38974662-SNV	2	38,974,662	4.5*	3.0 ns	0.7 ns	3.4 ns	100	T	0.41	T/A	No gene
2:43513959-SNV	2	43,513,959	4.0 ns	2.3 ns	1.1 ns	4.4*	100	C	0.49	T/C	*Glyma.02 g247400*
Salt-20	3	38,610,964	29.0*	25.6*	7.2*	27.4*	99.6	C	0.45	C/G	*Glyma03g32900* ^b^
Salt14056	3	38,619,995	32.4*	28.2*	8.2*	29.6*	100	G	0.46	C/G	*Glyma03g32900* ^b^
Salt11655	3	38,622,492	27.2*	24.1*	6.2*	25.7*	99.6	T	0.45	G/T	*Glyma03g32900* ^b^
5:2725777-SNV	5	2,725,777	1.1 ns	2.3 ns	5.5*	4.9*	100	T	0.50	T/C	*Glyma.05 g031300*
6:3536892-SNV	6	3,536,892	1.1 ns	2.3 ns	5.5*	4.9*	100	A	0.50	A/G	No gene
6:38850839-SNV	6	38,850,839	4.8*	2.9 ns	2.2 ns	5.2*	100	A	0.25	A/G	No gene
8:11762527-SNV	8	11,762,527	4.2 ns	3.7 ns	5.2*	6.6*	100	T	0.35	T/A	No gene
8:11859355-SNV	8	11,859,355	4.3*	3.4 ns	3.6 ns	5.3*	100	T	0.32	T/A	No gene
8:11869912-SNV	8	11,869,912	4.1 ns	3.3 ns	3.5 ns	5.0*	100	T	0.3	T/C	*Glyma.08 g153800*
8:11897542-SNV	8	11,897,542	4.1 ns	3.3 ns	3.3 ns	4.6*	100	C	0.3	C/T	*Glyma.08 g154400*
8:12168563-SNV	8	12,168,563	3.3 ns	3.5 ns	6.7*	4.4*	100	G	0.38	G/A	*Glyma.08 g157400*
8:12177874-SNV	8	12,177,874	2.9 ns	3.7 ns	6.9*	3.4 ns	100	A	0.48	A/T	*Glyma.08 g157400*
8:12198547-SNV	8	12,198,547	3.8 ns	4.1 ns	7.6*	4.6*	100	C	0.4	C/T	No gene
8:12221598-SNV	8	12,221,598	3.0 ns	2.8 ns	6.7*	4.3 ns	100	G	0.4	G/A	*Glyma.08 g157700*
8:18197526-SNV	8	18,197,526	3.5 ns	3.2 ns	3.1 ns	5.0*	100	T	0.07	T/C	*Glyma.08 g224400*
8:18363900-SNV	8	18,363,900	4.5*	3.6 ns	2.2 ns	4.6*	100	A	0.47	A/G	No gene
8:18378659-SNV	8	18,378,659	4.8*	4.5*	3.3 ns	3.7 ns	100	G	0.48	T/G	No gene
14:8102511-SNV	14	8,102,511	4.6*	3.7 ns	1.2 ns	2.2 ns	100	T	0.49	T/C	No gene
14:47627721-SNV	14	47,627,721	1.2 ns	0.9 ns	5.6*	0.9 ns	100	A	0.06	A/G	*Glyma.14 g211300*
15:11833367-SNV	15	11,833,367	3.0 ns	2.4 ns	3.4 ns	5.0*	100	C	0.49	C/T	*Glyma.15 g143900*
16:34944055-SNV	16	34,944,055	4.9*	4.1 ns	1.3 ns	2.4 ns	100	C	0.37	C/A	No gene
18:56065139-SNV	18	56,065,139	1.1 ns	2.3 ns	5.5*	4.9*	100	G	0.50	G/T	*Glyma.18 g294400*
18:57153685-SNV	18	57,153,685	3.2 ns	5.3*	0.8 ns	3.2 ns	100	T	0.23	T/C	No gene
18:57203235-SNV	18	57,203,235	4.9*	4.1 ns	2.5 ns	2.9 ns	100	A	0.17	A/T	No gene
19:43567289-SNV	19	43,567,289	7.5*	6.7*	2.1 ns	5.1*	100	T	0.20	T/G	*Glyma.19 g175600*
20:37261341-SNV	20	37,261,341	3.3 ns	4.6*	0.5 ns	1.6 ns	100	C	0.06	T/C	No gene
-log10(*P*) threshold ^c^			4.3	4.4	5.1	4.3					

*LSS* leaf scorch score, *CCR* chlorophyll content ratio, *LSC* leaf sodium content, *LCC* leaf chloride content (LCC)

*: Significant association with salt tolerant traits; ns: None significant association with trait

^a^: The candidate gene in Soybase Wm82 Genome Browser version 2 consisting of significant SNP; ^b^: The cloned gene for salt tolerance is named *Glyma03g32900* in SoyBase Wm82 Genome Browser version 1; ^c^: Threshold was calculated based on *P*-value using False Discovery Rate correction (Benjamini-Hochberg); MA: Minor allele; MAF: Minor allele frequency

### Putative candidate genes underlying salt tolerance

Phytozome and Soybase genome browsers (https://phytozome.jgi.doe.gov/jbrowse/ and https://soybase.org/gb2/gbrowse/) were used to detect additional genes in the significant genomic regions associated with salt tolerant traits in addition to the known gene on Chr. 3. The genomic intervals with the group of the significant SNPs ranged from 2788 to 787,140 bp and were considered significant genomic regions to search for putative candidate genes. A total of 222 genes were found in the significant genomic intervals (Additional file 8: Table S3) including the genes underlying the most significant SNPs on each chromosome (Table 3). Among these, 157 genes have predicted functions and known protein families in Phytozome and Soybase databases. Based on functional annotation, transporters (*Glyma.08 g146100, Glyma.08 g224400*), ion channel (*Glyma.02 g204300*), membrane proton pump (*Glyma.08 g225500*), universal stress protein (*Glyma.14 g211300*), and Callose synthase (*Glyma.08 g157400*) could be additional candidate genes related to salt tolerance in soybean. Four genes were located in two adjacent, significant genomic intervals surrounding the minor locus on Chr. 8 and others on Chrs. 2 and 14.

### Identification of new sources of salt tolerance

Soybean accessions belonging to salt tolerant and salt sensitive groups were classified by combining results of LSS, CCR, LSC and LCC (Fig. 2) and were tested with genotypes of three GBMs. To evaluate the genotype-phenotype association, genotypes of GBMs (Salt-20, Salt14056 and Salt11655) among 305 soybean accessions were classified and evaluated by compatibility with phenotypes. The GBM genotypes of salt tolerant checks (Fiskeby III and Lee) were classified as mutant (mut) alleles while those of salt sensitive checks (Hutcheson and Jackson) were wide-type (wt) alleles (Table 4). There was a strong genotype-phenotype association in the diverse panel of soybean accessions. Over 90% of the accessions had a perfect genotype-phenotype match, in which salt tolerant and salt sensitive lines carried mutant and wide-type alleles of all three GBMs, respectively. There were some salt sensitive lines with recombination of the three GBMs; however, no salt sensitive lines carried mutant alleles of all three GBMs. In the salt tolerant group, 9 lines had a combination of three GBMs (Table 4), suggesting that these soybean accessions may be new salt tolerant sources with new alleles of the known salt tolerant gene on Chr. 3. Additionally, another six lines without mutant alleles of the three GBMs (Table 4) were predicted as salt tolerant sources carrying new gene(s) for salt tolerance in soybean. Additional studies are need to confirm such predictions.

**Table 4:** New sources for salt tolerance from matching analysis of phenotype and gene-based marker genotypes

PI	Taxonomy	Phenotypic Group	Genotypes of GBMs			Prediction/Suggestion
			Salt-20	Salt14056	Salt11655	
PI597458B	*G. soja*	Tolerant	Wt	Mut	Mut	New allele on Chr. 3
PI342434	*G. max*	Tolerant	Wt	Mut	Wt	New allele on Chr. 3
PI548198	*G. max*	Tolerant	Wt	Mut	Wt	New allele on Chr. 3
PI561389B	*G. max*	Tolerant	Wt	Mut	Wt	New allele on Chr. 3
PI407202	*G. soja*	Tolerant	Wt	Mut	Wt	New allele on Chr. 3
PI407220	*G. soja*	Tolerant	Wt	Mut	Wt	New allele on Chr. 3
PI424107A	*G. soja*	Tolerant	Wt	Mut	Wt	New allele on Chr. 3
PI479752	*G. soja*	Tolerant	Wt	Mut	Wt	New allele on Chr. 3
PI407083	*G. soja*	Tolerant	Wt	Wt	Mut	New allele on Chr. 3
PI468908	*G. max*	Tolerant	Wt	Wt	Wt	New gene
PI080837	*G. max*	Tolerant	Wt	Wt	Wt	New gene
PI417500	*G. max*	Tolerant	Wt	Wt	Wt	New gene
PI424116	*G. soja*	Tolerant	Wt	Wt	Wt	New gene
PI483460B	*G. soja*	Tolerant	Wt	Wt	Wt	New gene
PI562551	*G. soja*	Tolerant	Wt	Wt	Wt	New gene
PI438471	*G. max*	Tolerant check	Mut	Mut	Mut	
PI548656	*G. max*	Tolerant check	Mut	Mut	Mut	
PI518664	*G. max*	Sensitive check	Wt	Wt	Wt	
PI548657	*G. max*	Sensitive check	Wt	Wt	Wt	

Wt: Wide-type allele of GBMs (Salt-20, Salt14056, Salt11655)

Mut: Mutant allele of GBMs (Salt-20, Salt14056, Salt11655)

## Discussion

The association mapping approach is suited for the detection and characterization of quantitative traits because of broad genetic base of natural populations [27–29]. The development of statistical models and multiple tests to control effects of population structure and relatedness [34–37], as well as exploration of whole-genome sequencing data, has made GWAS more efficient in identifying the significant genomic regions associated with the traits of interest and in predicting candidate genes [45, 48]. In this study, both SoySNP50K- and WGRS-derived SNP datasets were sufficient to cover the genome-wide haplotype blocks. There was no genomic inflation by population stratification found in the association analysis using EMMAX and MLMM (Additional file 5: Figure S3 and Additional file 7: Figure S5; Additional file 4: Table S2). Nevertheless, GWAS for detecting salt tolerance using the SoySNP50K dataset only confirmed the major locus on Chr. 3. On the other hand, using the 3.7 M SNP dataset discovered more significant genomic regions, including a minor locus on Chr. 8. Additionally, three GBMs of the known gene (*Glyma03g32900*) on Chr. 3 [17, 19, 21] were significantly associated with salt tolerance by SoySNP50K-based GWASs (Fig. 4b) while there were many other significant SNPs in the known gene identified by WGRS-based GWASs (Additional file 9: Figure S6). Pinpointing the known gene was only accomplished by integration of GBMs into the SoySNP50K dataset. Therefore, a larger SNP dataset generated from whole-genome sequencing was better for GWAS to detect new loci for salt tolerance.

The major locus on Chr. 3, in which the known gene for salt tolerance (*Glyma03g32900*) was confirmed by previous GWAS [23, 50]. By integrating the three GBMs into the SNP marker datasets, the known gene was pinpointed with the most significant SNPs associated with all four traits by both SoySNP50K- and WGRS-based GWAS (Tables 2 and 3; Figs. 4a and 5a). Also, the other genomic regions associated with four traits have been mapped by association mapping in previous studies. A GWAS analysis of 191 landraces for three germination-related traits under high salt conditions using 1142 SNPs determined 13 SNPs associated with salt tolerance on Chrs. 2, 3, 6, 8, 9, 12, 13, 14, 17 and 18 and were reported with a log10(*P*) range of 2.05–3.60 by using a mixed linear model [49]. In a previous association study of a panel of 283 soybean lines with 33,009 SNPs (SoySNP50K dataset), Zeng et al. [50] evaluated two traits, leaf chloride concentrations and leaf chlorophyll concentrations, and confirmed the major locus on Chr. 3 and detected additional genomic regions on Chrs. 2, 7, 8, 10, 13, 14, 16 and 20 with -log10(*P*) > 4.1 and -log10(*P*) > 2.1 thresholds for a GLM and MLM, respectively. The results of those association mapping studies require a confirmation because of -log10 (*P*) thresholds to control spurious association did not meet the requirements for GWAS [49, 50]. In our study, the significant SNPs associated with salt tolerance using both SNP marker datasets were found on 15 chromosomes based on the Benjamini-Hochberg correction with FDR ≤ 0.05 (Tables 2 and 3). Consequentially, the major locus on Chr. 3, minor locus on Chr. 8, and significant genomic regions on Chrs. 1 and 18 were confirmed and identified by a combination an association of salt tolerant traits and results of GWAS using MLMM. Comparing to previous studies, the minor locus on Chr. 8 identified in our study was 1,449,275 bp away from BARC-041663-08059 associated with germination-related traits under salt stress [49] and 17,932,879 bp away from ss715601563 associated with salt tolerance [50]. Thus, the significant genomic region on Chr. 8 associated with salt tolerance in this study strongly predicts a new minor locus for salt tolerance in soybean.

In addition, a Cl^−^-tolerant QTL located between 27,665,585 and 28,206,014 bp on Chr. 13 was identified by linkage mapping of KCl-salt tolerance [25]. Another QTL for leaf sodium content was mapped between 38,366,685 and 40,167,119 bp on Chr. 13 [20]. In our study only one SNP marker, ss715616720, was significantly associated with leaf scorch score and chlorophyll content ratio (Table 2) from SoySNP50K-based GWAS using EMMAX. However, this SNP marker was undetected after one step of MLMM and WGRS-based GWAS which could be a spurious association due to confounding between testing markers and kinship [36]. Thus, the locus for salt tolerance on Chr. 13, which was not identified in this study, may carry rare salt tolerant alleles at low frequency in the diverse panel.

As efficient tools for candidate gene prediction, Phytozome and Soybase genome browsers (https://phytozome.jgi.doe.gov/jbrowse/ and https://soybase.org/gb2/gbrowse/) provided a mechanism to search and predict putative candidate genes for iron deficiency chlorosis [51], nitrogen fixation traits [52], soybean seed germination under salt stress [49], sudden death syndrome resistance [53], *Phytophthora sojae* resistance [54], and soybean cyst nematode resistance [55, 56]. Using a similar approach, *Glyma.08 g146100* (EamA-like transporter family), *Glyma.08 g157400* (SF9 - Callose synthase), *Glyma.08 g224400* (V-type H^+^-transporting ATPase subunit A), and *Glyma.08 g225500* (SF11 - Pyrophosphate-energized membrane proton pump) were predicted as putative candidate genes for salt tolerance that was mapped on Chr. 8 by functional nominations. Similar functional genes have been reported controlling salt tolerance or abiotic stress in plants, such as Callose synthase which plays an important role in response to multiple biotic and abiotic stresses, including salt stress [57, 58]. In addition, V-type H^+^-transporting ATPase subunit expressed in the roots, energizes sodium sequestration into the central vacuole and enhances salt tolerance in plants [59–61]. Therefore, the putative candidate genes should be considered for post-GWAS analysis such as gene expression.

The association between salt tolerance and structure of the known gene (*Glyma03g32900*) on Chr. 3 [19, 23] or gene profiling expression of this gene [21] suggests other salt tolerant sources with novel genes for salt tolerance. Three GBMs, Salt-20, Salt14056 and Salt11655, based on the *Glyma03g32900* sequence showed a high association with salt tolerance [20, 23] and were confirmed in this study. However, a few salt tolerant lines were not a genotype-phenotype match that carried salt sensitive alleles of these GBMs. The results suggest that those salt tolerant lines might have new salt tolerant allele(s) of the Chr. 3 known locus and other lines might carry novel gene(s) for soybean salt tolerance.

## Conclusions

The salt tolerant traits investigated and multiple genomic regions and SNPs identified in this study confirm that salt tolerance is a complex trait with multiple genes and mechanisms involved. The SNP dataset developed from the WGRS was more efficient than those of the SoySNP50K dataset for GWAS to predict minor loci and to pinpoint putative candidate genes for salt tolerance in soybean. The significant genomic regions strongly suggested a minor locus associated with salt tolerance on Chr. 8. The putative candidate genes and the significant SNPs may be useful to study the molecular mechanism and for marker-assisted selection and molecular breeding to improve salt tolerance in soybean. Additionally, six new salt tolerant sources with predicted novel gene(s) should be further investigated to identify additional salt tolerant gene(s). Moreover, using LSS and/or CCR would be simple and efficient in selection for salt tolerance in a practical breeding program as measuring LSC and LCC is time consuming and expensive and the four traits are highly correlated.

## Methods

### Plant materials

A core set of 305 soybean plant introductions (PIs) was selected from the USDA Soybean Germplasm Collection representing wide genetic diversity derived from the SoySNP50K iSelect BeadChip analysis (Drs. Cregan and Song, pers. comm.). This diverse panel had 255 cultivated soybean (*Glycine max*) and 50 wild soybean (*Glycine soja*) accessions. In addition, two salt-tolerant genotypes, cultivars Lee and Fiskeby III [20, 62–64], two salt-sensitive genotypes, cultivars Hutcheson and Jackson [16, 18], and cultivar, Williams 82, were also included as checks. According to the Germplasm Resources Information Network-National Plant Germplasm System (GRIN, *https://www.ars-grin.gov/npgs*) database, this core set contains accessions from maturity group (MG) 000 to X and originated from 28 different countries (Additional file 10: Table S4). While conducting GWAS, a subset of 234 accessions from this germplasm set was used for a separate association analysis based on the availability of the WGRS-derived 3.7 M SNP dataset (*http://www.soybase.org*).

### Genotypic datasets

Over 42,000 SNP markers from the Illumina Infinium SoySNP50K iSelect BeadChip [65] were accessed from the soybean database (*http://www.soybase.org*). Of these, a total of 37,573 SNPs was selected based on the exclusion of SNPs with greater than 5% missing data and a minor allele frequency (MAF) of less than 5%. A second SNPs dataset of over 4.7 M SNPs was generated from the United Soybean Boarded-funded whole-genome resequencing (WGRS) project in the Nguyen Lab (*http://www.soybase.org*). This SNP dataset was further filtered based on the exclusion of the missing data (> 5%) and MAF (< 5%) to obtain over 3.7 M qualified SNP using the SNP Variation & Suites (SVS) program (Golden Helix, Bozeman, MT 59718, USA). This SNP dataset is hereafter referred as the WGRS-derived 3.7 M SNP dataset. Additionally, three GBMs, Salt-20, Salt14056 and Salt11655, that were previously reported [20, 23] were also incorporated into these SNP marker datasets.

### Phenotyping

Soybean lines of the diverse panel were evaluated for salt tolerance under greenhouse conditions following a previously described method [66] with minor modifications. The experimental design was a randomized block with 3 replications blocked over time. Five seedlings of each line were grown per cone-tainer and evaluated in a greenhouse at the University of Missouri, Columbia, MO, using artificial lights and a 13 h photoperiod from September to December 2016. Soybean seedlings at the growth stage V2 [67] were treated with salt water by exposing seedlings in cone-tainers to a 120 mM NaCl solution in a tank. The salt solution in the tank was kept at one-third (7 cm) from the bottom of cone-tainers for 7 h/day. When the salt-sensitive checks showed severe leaf scorch, which typically appears approx. 2 weeks after the treatment, leaf scorch score (LSS) was visually scored for each plant using a 1–5 scale, where 1 = no apparent chlorosis; 2 = slight (25% of the leaves showed chlorosis); 3 = moderate (50% of the leaves showed chlorosis and some necrosis); 4 = severe chlorosis (75% of the leaves showed chlorosis and severe necrosis); and 5 = dead (leaves showed severe necrosis and were withered), mean of LSS of each line was then calculated as previously described [66]. Leaf chlorophyll content was measured for the topmost fully expanded leaf 1 day before and about 14 days after the salt treatment using a chlorophyll meter (Chlorophyll meter SPAD-502, Konica Minolta, Inc., Osaka, Japan) to calculate chlorophyll content ratio (CCR) [20].

Combined trifoliate leaves of five seedlings from each soybean line were harvested after being read for leaf scorch score and chlorophyll content and dried at 60 °C for 7 days. The dried leaf tissue was ground using a Thomas Model ED-5 laboratory Wiley mill (Thomas Scientific, Swedesboro, NJ, USA) for analyzing leaf chloride (LCC) and leaf sodium (LSC) contents at the Delta Research Center, University of Missouri, Portageville, MO [20, 64]. Briefly, sodium concentration was determined by processing ground leaf tissue (0.25 g) with a modified wet acid dilution and measuring in an atomic absorption spectrophotometer (Perkin-Elmer™, Wellesley, MA, USA). Standard reference curves were established for calibration of 25, 50, 100 and 500 mg kg^− 1^ of chloride. Then chloride concentration in solution of 0.15 g ground leaf tissue was measured using an ion specific electrode in a Fisher Scientific AR 50 dual channel pH, ion, conductivity-meter (Fischer Scientific, Pittsburg, PA, USA). Finally, sodium and chloride concentrations were converted to mg per kg of dry leaf tissue for leaf sodium content (LSC) and leaf chloride content (LCC).

### Phenotypic data analysis

Analysis of variance (ANOVA) and the estimation of variance components of phenotypic data were performed using the PROC GLM procedure of SAS 9.4 [68]. Broad-sense heritability (H^2^) of four salt tolerance associated traits were calculated using the following equation for randomized block design: H^2^ = σ^2^_g_/(σ^2^_g_ + σ^2^_e_), where σ_g_ is the genotypic variance and σ_e_ is the error variance [69, 70]. Soybean lines were grouped into salt tolerant and salt sensitive groups based on combining all four salt tolerant traits using Euclidean distances in NTSYSpc 2.1 [71]. In addition, the Pearson correlations were also calculated to measure the degree of linear relationship between each pairs of traits and the individual hypothesis tests of the correlations were performed at α = 0.01 using Minitab 17 [72].

### Genome-wide association study (GWAS)

Linkage disequilibrium (LD) and correlation coefficients (*r*^*2*^) were measured on all adjacent pairs within each chromosome and a fitted curve was computed based on nonlinear regression of LD (*r*^*2*^) on distance (kb) as previously defined [73]. Principle components (PCs) and kinship matrix (relatedness) were applied to correct for population structure and relatedness in mixed linear models. Principal component analysis (PCA) was done for all filtered SNPs with MAF > 0.05 and the call rate > 0.95. Number of PCs were selected to add in models when an inflation factor (λ) of *p*-value was close to one [74, 75] using a genotype association test with a PCs correction in SNP & Variation Suite (SVS) v8.7.0 software (Golden Helix, Bozeman, MT, USA). Identity by state (IBS) matrices indicated relatedness among soybean accessions calculated using TASSEL 5 [76] to construct phylogenic trees in MEGA 7 [77] and to apply as kinship matrix in GWAS. Genome-wide associations between SNPs and salt tolerant trait were identified using the efficient mixed-model association expedited (EMMAX) and multi-locus mixed model (MLMM) with correction for population structure and relatedness in the SVS software (Golden Helix, Bozeman, MT, USA). False positives were controlled by multiple test correction with false discovery rate (FDR) ≤ 0.05 [78] and the threshold of -log10(*p*-value) for identifying significant associations was calculated at FDR = 0.05. The significant SNPs associated with salt tolerant traits were counted with larger -log10(*p*-value) than the threshold that was calculated based on *P*-value using the False Discovery Rate correction (Benjamini-Hochberg) and causal SNPs surrounding the known gene for salt tolerance on Chr. 3 [17, 19, 21].

### Candidate genes of salt tolerance

The soybean genomic data, the soybean genome assembly (Wm82.a2.v1), was integrated with the genomic scale data visualization tool by importing to the GWAS project created in the SVS software (Golden Helix, Bozeman, MT, USA). The search for candidate genes was conducted by zooming in at positions of the significant SNPs associated with salt tolerant traits. Information about the candidate gene was displayed by automatically linking with the Genome Browser of phytozome website (https://phytozome.jgi.doe.gov/jbrowse/). The results were double checked by searching the SoyBase Wm80 Genome Browser (https://soybase.org/gb2/gbrowse/).

### Identification of new sources of salt tolerance using gene-based markers

The soybean lines from salt tolerant group based on their phenotypes were selected to evaluate matching genotypes of GBMs. The salt tolerant lines that did not match the genotypes of GBMs were identified as new sources that may carry new salt tolerant gene(s) in addition to the known gene on Chr. 3 [17, 19, 21]. Moreover, recombination between three GBMs in the salt tolerant lines was considered as new allele(s) of the locus on Chr. 3 for salt tolerance.

## Additional files

Additional file 1: **Table S1.** Variation of salt tolerance of checks grown under 120 mM NaCl treatment. (DOCX 12 kb)

Additional file 2: **Figure S1.** Comparisons of salt tolerance based on leaf scorch between soybean cultivars. Fiskeby III and Lee (salt tolerant checks), Hutcheson and Jackson (salt sensitive checks), Williams 82 (the soybean reference cultivar). (JPG 6832 kb)

Additional file 3: **Figure S2.** Linkage disequilibrium (LD) decay plot of coefficient of correlation (*r*^*2*^) between adjacent marker pairs and genomic distance (kb). The fitted curves are based on nonlinear regression using SoySNP50K dataset from 305 diverse genotypes (*yellow*) and using 3.7 M SNP dataset in the subset from 234 genotypes selected from the original 305 accessions (*blue*). (JPG 1168 kb)

Additional file 4: **Table S2.** Genomic inflation factor (λ) of models for analyzing association with salt tolerance among soybean accessions using both datasets. (DOCX 13 kb)

Additional file 5: **Figure S3.** Quantile-quantile (Q-Q) plots showing the expected -log10(*P*) compared to the observed -log10(*P*). The results of statistical testing (EMMAX) for association across 37,573 SNPs from SoySNP50K dataset with leaf scorch score (A), chlorophyll content ratio (B), leaf sodium content (C) and leaf chloride content (D) among 305 genetically diverse soybean genotypes. Most SNPs matched with solid lines [expected -log10(*P*) = observed -log10(*P*)] were unassociated SNPs, on the other hand, sharp curves at the end are the number of true associations. (JPG 4403 kb)

Additional file 6: **Figure S4.** Manhattan plots showing association of SNPs distributed throughout 20 chromosomes with four traits. The results based on analyzing GWAS of leaf scorch score (LSS), chlorophyll content ratio (CCR), leaf sodium content (LSC) and leaf chloride content (LCC) using 37,573 SNPs from SoySNP50K dataset from the subset of 234 diverse accessions selected from the original 305 accessions. (JPG 2008 kb)

Additional file 7: **Figure S5.** Quantile-quantile (Q-Q) plots showing the expected -log10(*P*) compared to the observed -log10(*P*). The results of statistical testing (EMMAX) for association across 2,280,225 polymorphic SNPs from 3.7 M SNP dataset with leaf scorch score (A), chlorophyll content ratio (B), leaf sodium content (C) and leaf chloride content (D) in the subset of 234 soybean lines selected from the original population of 305 genotypes. SNPs matched with solid lines [expected -log10(*P*) = observed -log10(*P*)] were unassociated SNPs, on the other hand, sharp curves at the end presented the number of true associations. (JPG 4813 kb)

Additional file 8: **Table S3.** The genes underlying the significant genomic regions associated with salt tolerance from Phytozome and Soybase databases. (XLSX 24 kb)

Additional file 9: **Figure S6.** The significant SNPs underlying the known gene (*Glyma03g32900*) on Chr. 3 (*red dots*) associated with salt tolerance. The known gene (*Glyma03g32900*) named in SoyBase Wm82 Genome Browser version 1, and LD block in this genomic region. (JPG 647 kb)

Additional file 10: **Table S4.** A core set of 305 soybean plant introductions (PIs) selected from the USDA Soybean Germplasm Collection. (XLSX 27 kb)

## Abbreviations

CCR: Chlorophyll content ratio
GBM: Gene-based marker
GWAS: Genome-wide association study
LCC: Leaf chloride content
LD: Linkage disequilibrium
LSC: Leaf sodium content
LSS: Leaf scorch score
NGS: Next-generation sequencing
PCA: Principal component analysis
QTL: Quantitative trait loci
SNP: Single nucleotide polymorphism
WGRS: Whole-genome resequencing
