# Supplementary material for: Identification of new loci for salt tolerance in soybean by high-resolution genome-wide association mapping
Source: BMC Genomics. 2019 Apr 25;20:318. doi: 10.1186/s12864-019-5662-9 (PMC6485111; doi:10.1186/s12864-019-5662-9)
Supplement: Supplementary file 1 — Table S1. Variation of salt tolerance of checks grown under 120 mM NaCl treatment. (DOCX 12 kb) [file 12864_2019_5662_MOESM1_ESM.docx]

**Table S1.** **Variation of salt tolerance of checks grown under 120 mM NaCl treatment.**

| **Line** | **LSS^a^** | **CCR^b^** | **LSC** (g kg^-1^) | **LCC** (g kg^-1^) |
| --- | --- | --- | --- | --- |
| Lee (tolerant check) | 1.8±0.2 | 1.0±0.03 | 0.4±0.1 | 5.9±1.3 |
| Fiskeby III (tolerant check) | 1.0±0.1 | 1.1±0.01 | 0.2±0.1 | 4.9±0.1 |
| Hutcheson (sensitive check) | 4.3±0.1 | 0.6±0.01 | 0.6±0.1 | 9.8±1.0 |
| Jackson (sensitive check) | 4.8±0.1 | 0.5±0.04 | 0.9±0.2 | 12.8±2.1 |

LSS: leaf scorch score; CCR: chlorophyll content ratio; LSC: leaf sodium content; LCC: leaf chloride content; ^a^: Leaf scorch score based on a 1-5 scale; ^b^: The ratio of leaf chlorophyll content after treatment dividing leaf chlorophyll content after treatment
