# Supplementary figures and images for: Identification of new loci for salt tolerance in soybean by high-resolution genome-wide association mapping
Source: BMC Genomics. 2019 Apr 25;20:318. doi: 10.1186/s12864-019-5662-9 (PMC6485111; doi:10.1186/s12864-019-5662-9)

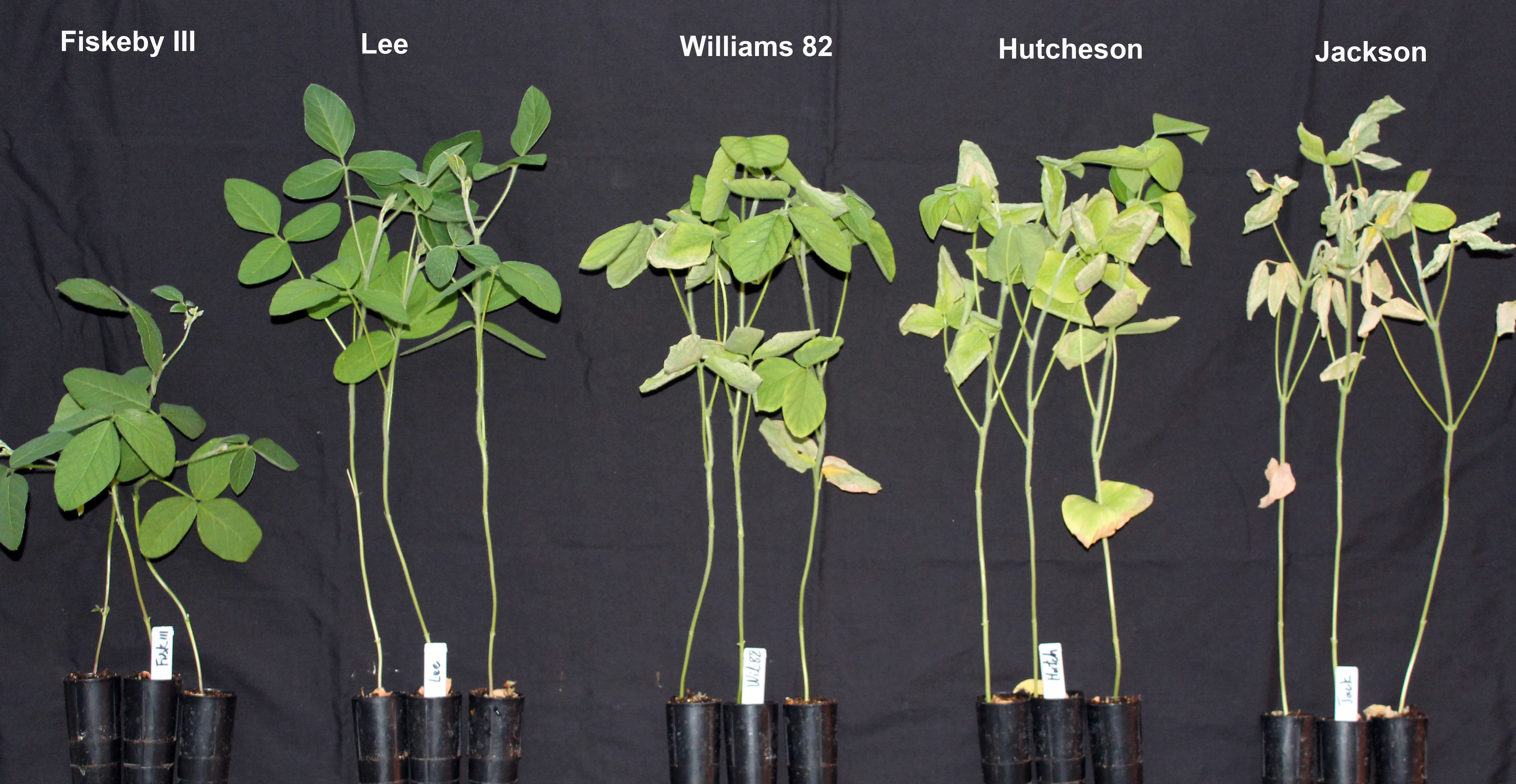

Supplement: Supplementary file 2 — Figure S1. Comparisons of salt tolerance based on leaf scorch between soybean cultivars. Fiskeby III and Lee (salt tolerant checks), Hutcheson and Jackson (salt sensitive checks), Williams 82 (the soybean reference cultivar). (JPG 6832 kb) [file 12864_2019_5662_MOESM2_ESM.jpg]

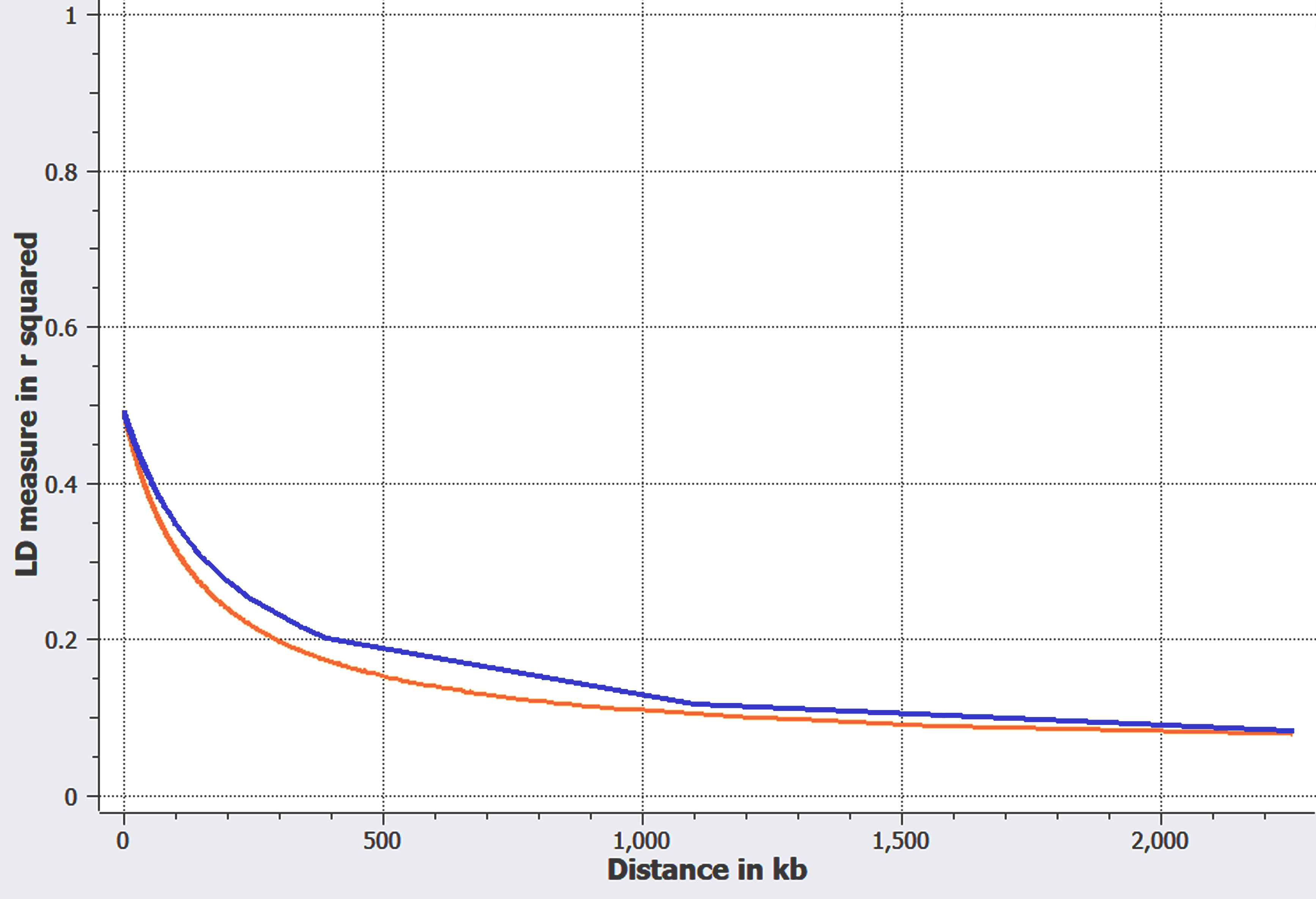

Supplement: Supplementary file 3 — Figure S2. Linkage disequilibrium (LD) decay plot of coefficient of correlation (r2) between adjacent marker pairs and genomic distance (kb). The fitted curves are based on nonlinear regression using SoySNP50K dataset from 305 diverse genotypes (yellow) and using 3.7 M SNP dataset in the subset from 234 genotypes selected from the original 305 accessions (blue). (JPG 1168 kb) [file 12864_2019_5662_MOESM3_ESM.jpg]

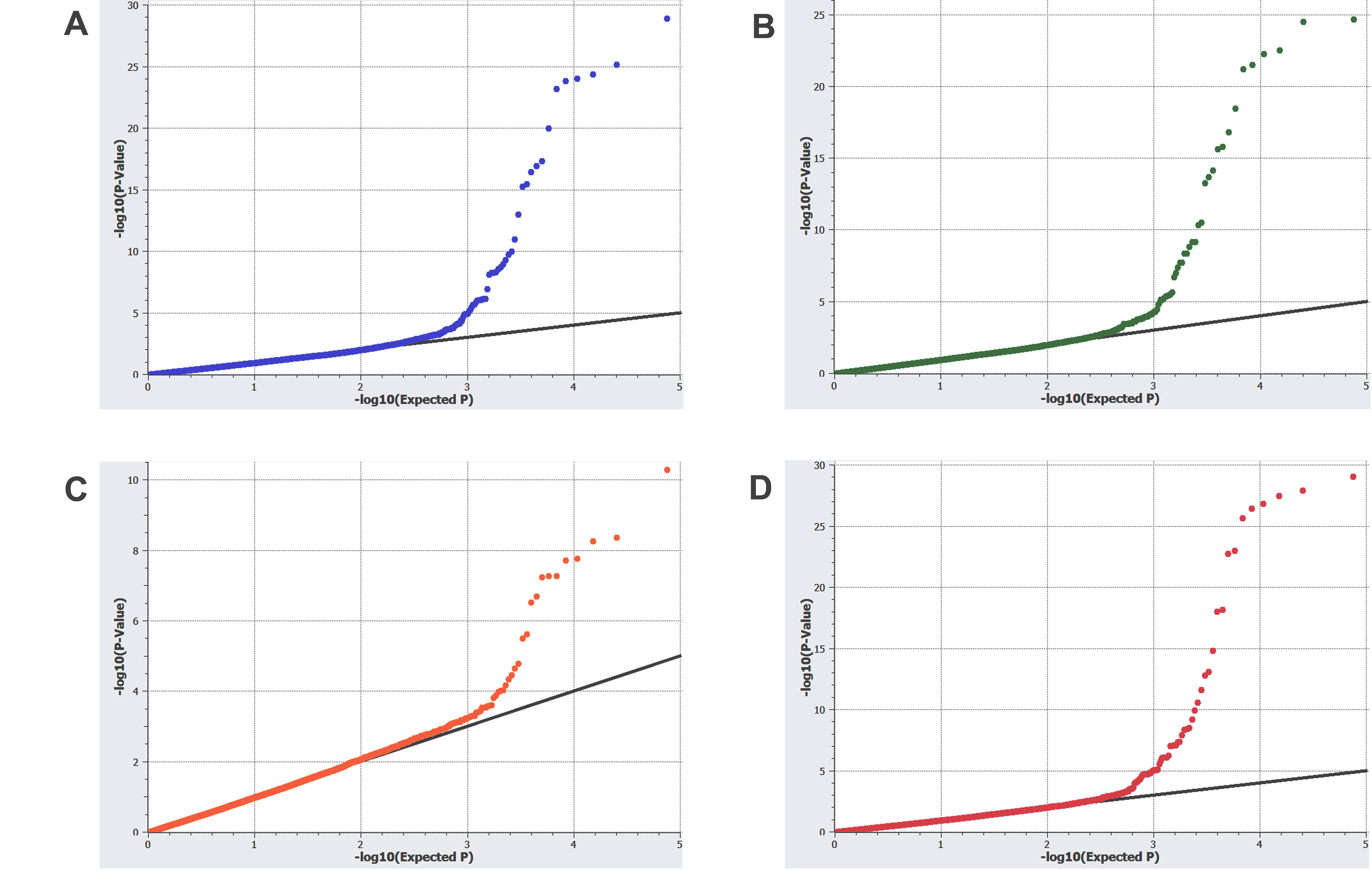

Supplement: Supplementary file 5 — Figure S3. Quantile-quantile (Q-Q) plots showing the expected -log10(P) compared to the observed -log10(P). The results of statistical testing (EMMAX) for association across 37,573 SNPs from SoySNP50K dataset with leaf scorch score (A), chlorophyll content ratio (B), leaf sodium content (C) and leaf chloride content (D) among 305 genetically diverse soybean genotypes. Most SNPs matched with solid lines [expected -log10(P) = observed -log10(P)] were unassociated SNPs, on the other hand, sharp curves at the end are the number of true associations. (JPG 4403 kb) [file 12864_2019_5662_MOESM5_ESM.jpg]

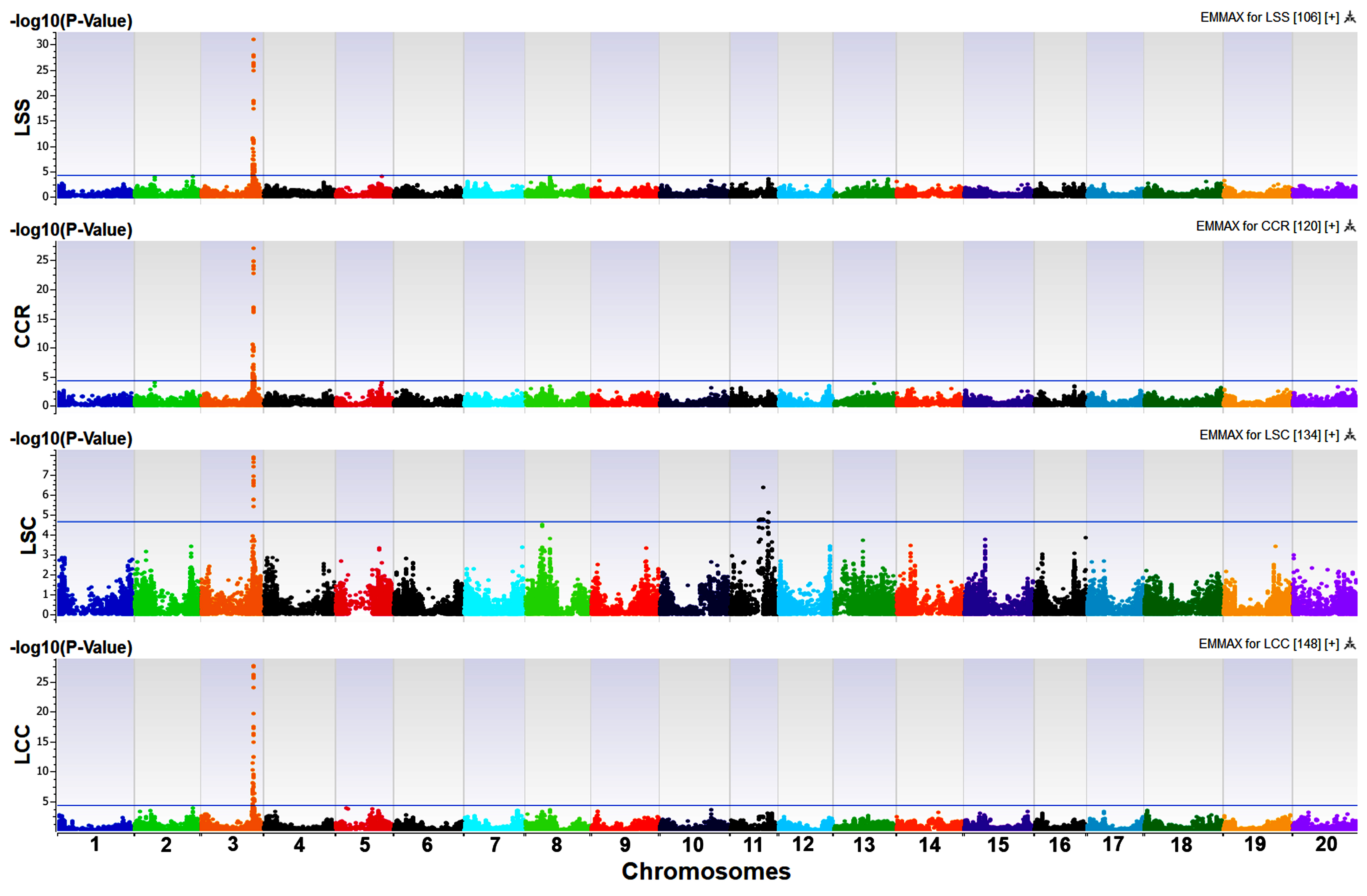

Supplement: Supplementary file 6 — Figure S4. Manhattan plots showing association of SNPs distributed throughout 20 chromosomes with four traits. The results based on analyzing GWAS of leaf scorch score (LSS), chlorophyll content ratio (CCR), leaf sodium content (LSC) and leaf chloride content (LCC) using 37,573 SNPs from SoySNP50K dataset from the subset of 234 diverse accessions selected from the original 305 accessions. (JPG 2008 kb) [file 12864_2019_5662_MOESM6_ESM.jpg]

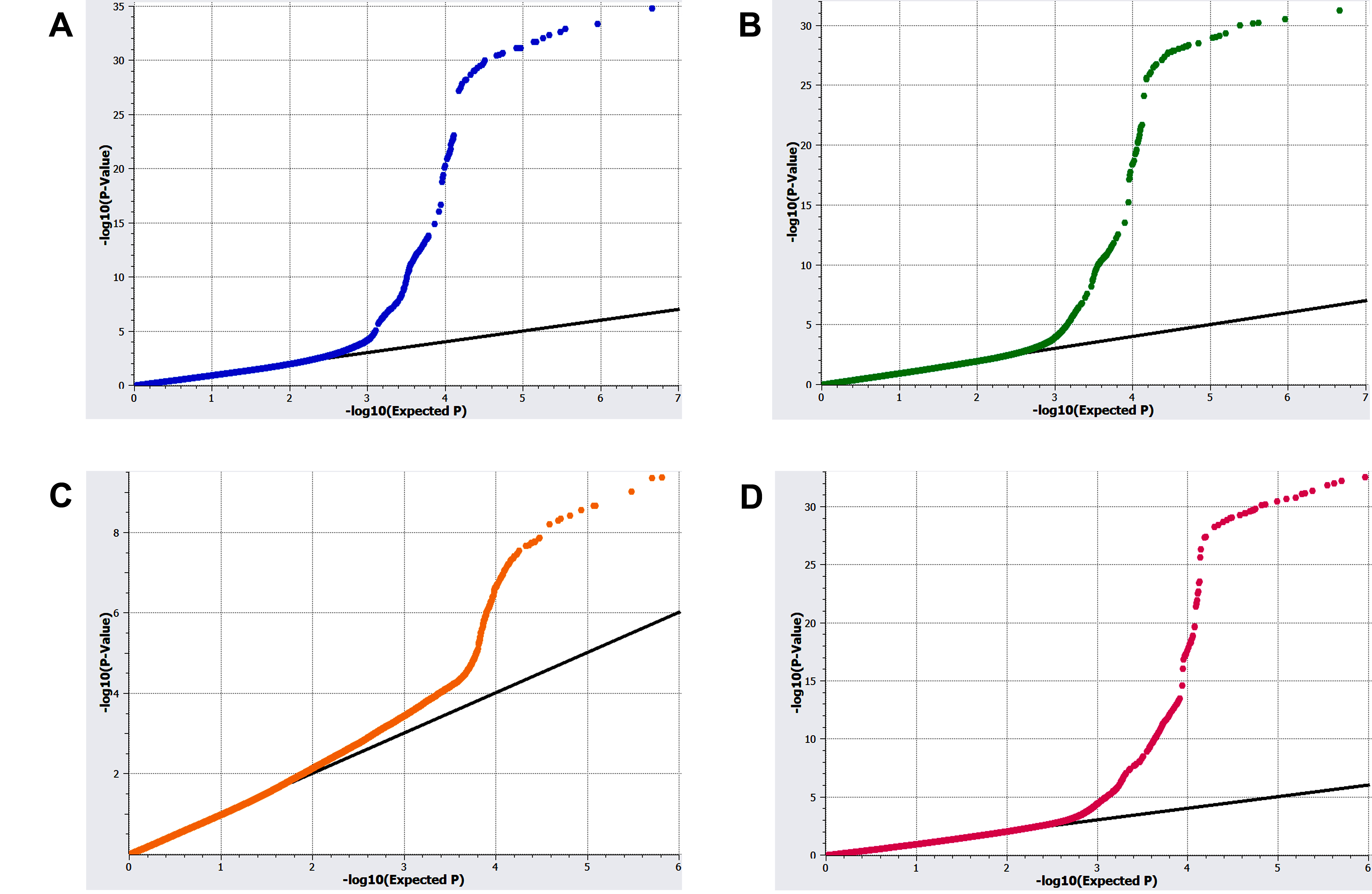

Supplement: Supplementary file 7 — Figure S5. Quantile-quantile (Q-Q) plots showing the expected -log10(P) compared to the observed -log10(P). The results of statistical testing (EMMAX) for association across 2,280,225 polymorphic SNPs from 3.7 M SNP dataset with leaf scorch score (A), chlorophyll content ratio (B), leaf sodium content (C) and leaf chloride content (D) in the subset of 234 soybean lines selected from the original population of 305 genotypes. SNPs matched with solid lines [expected -log10(P) = observed -log10(P)] were unassociated SNPs, on the other hand, sharp curves at the end presented the number of true associations. (JPG 4813 kb) [file 12864_2019_5662_MOESM7_ESM.jpg]

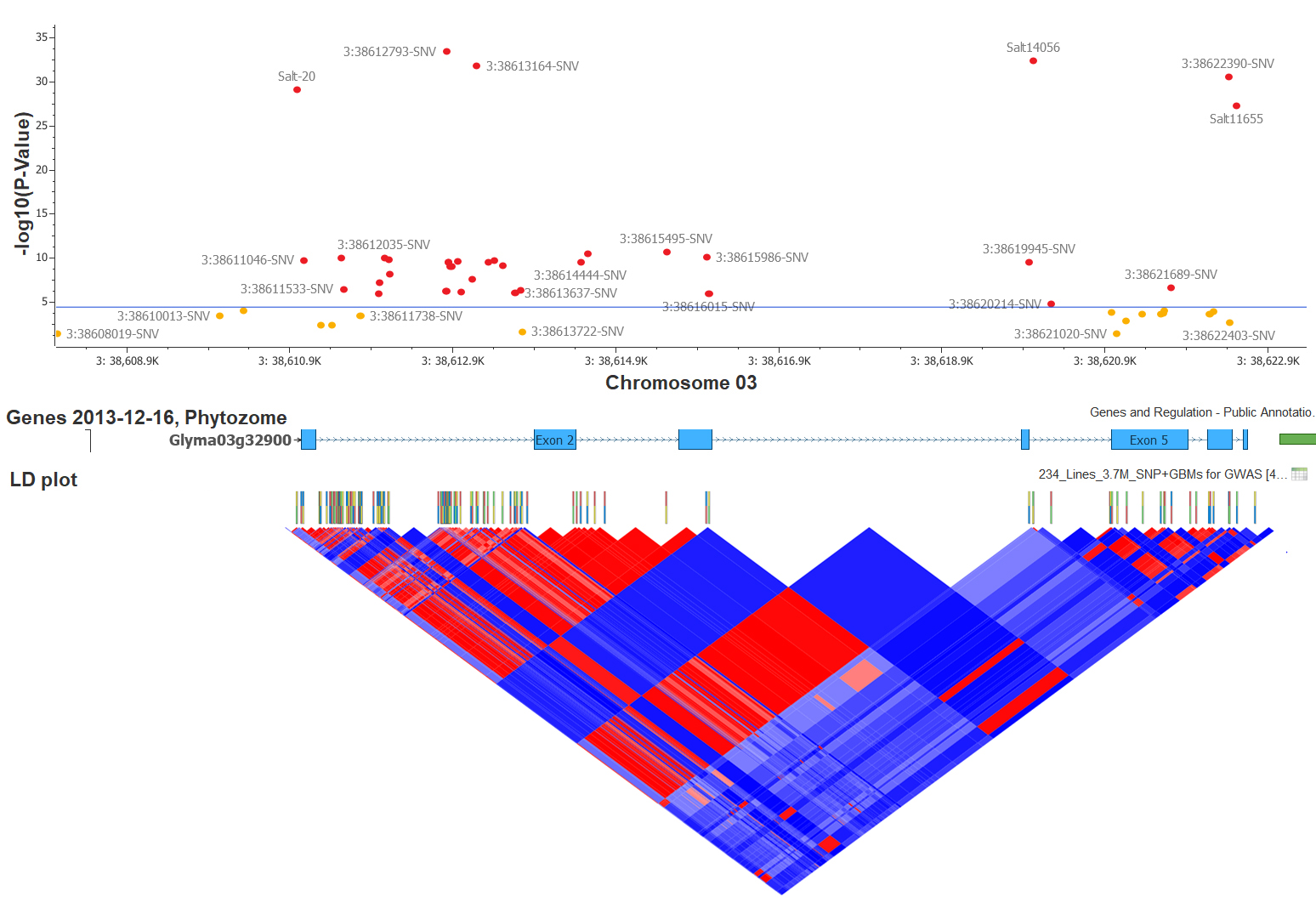

Supplement: Supplementary file 9 — Figure S6. The significant SNPs underlying the known gene (Glyma03g32900) on Chr. 3 (red dots) associated with salt tolerance. The known gene (Glyma03g32900) named in SoyBase Wm82 Genome Browser version 1, and LD block in this genomic region. (JPG 647 kb) [file 12864_2019_5662_MOESM9_ESM.jpg]
