# Supplementary material for: Identification of new loci for salt tolerance in soybean by high-resolution genome-wide association mapping
Source: BMC Genomics. 2019 Apr 25;20:318. doi: 10.1186/s12864-019-5662-9 (PMC6485111; doi:10.1186/s12864-019-5662-9)
Supplement: Supplementary file 4 — Table S2. Genomic inflation factor (λ) of models for analyzing association with salt tolerance among soybean accessions using both datasets. (DOCX 13 kb) [file 12864_2019_5662_MOESM4_ESM.docx]

**Table S2.** **Genomic inflation factor (λ) of models for analyzing association with salt tolerance among soybean accessions using both datasets**

| **Dataset** | **Population** | **Trait** | **Naïve** (λ) | **P model** (λ) | **EMMAX** (λ) | **The step of MLMM** (λ) | | | | |
| --- | --- | --- | --- | --- | --- | --- | --- | --- | --- | --- |
|  |  |  |  |  |  | **1** | **2** | **3** | **4** | **5** |
| SoySNP50K | Diverse panel (n=305) | LSS | 2.43 | 1.07 | 0.94 | 0.99 | 1.00 | 0.99 | 1.00 | 1.00 |
|  |  | CCR | 3.95 | 1.10 | 0.95 | 0.99 | 1.00 | 1.00 | 0.98 | 0.98 |
|  |  | LSC | 2.42 | 1.08 | 0.97 | 0.98 | 0.97 | 0.98 | 0.98 | 0.97 |
|  |  | LCC | 2.21 | 1.16 | 0.95 | 1.01 | 1.00 | 0.99 | 0.98 | 0.98 |
| 3.7M SNPs | Subset (n=234) | LSS | 1.74 | 1.15 | 0.95 | 0.98 | 1.00 | 1.00 | 1.01 | 1.03 |
|  |  | CCR | 1.98 | 1.20 | 0.97 | 0.99 | 1.02 | 0.94 | 0.95 | 0.96 |
|  |  | LSC | 1.44 | 1.05 | 0.97 | 0.99 | 1.00 | 1.01 | 1.00 | 1.02 |
|  |  | LCC | 2.08 | 1.17 | 0.93 | 0.98 | 0.98 | 0.98 | 0.98 | 0.96 |

LSS: leaf scorch score; CCR: chlorophyll content ratio; LSC: leaf sodium content; LCC: leaf chloride content; Naïve: The general linear model; P model: The statistical model with correction for principal components; EMMAX: Efficient mixed-model association expedited; MLMM: Multi-locus mixed model
